# The Relationship Between Pharyngeal Constriction and Post-swallow Residue

**DOI:** 10.1007/s00455-015-9606-5

**Published:** 2015-04-29

**Authors:** Shauna L. Stokely, Melanie Peladeau-Pigeon, Chelsea Leigh, Sonja M. Molfenter, Catriona M. Steele

**Affiliations:** 1 Toronto Rehabilitation Institute, University Health Network, 550 University Avenue, 12-101, Toronto, ON M5G 2A2 Canada; 2 Trillium Health Partners, Mississauga, Canada; 4 Communicative Sciences and Disorders Department, New York University, New York, USA; 3 University of Toronto, Toronto, Canada; 5 Bloorview Research Institute, Holland Bloorview Kids Rehabilitation Hospital, Toronto, Canada

**Keywords:** Dysphagia, Deglutition, Deglutition disorders, Videofluoroscopy, Pharyngeal constriction, Post-swallow residue

## Abstract

Pharyngeal constriction has been proposed as a parameter that may distinguish functional from impaired swallows. We employed anatomically normalized pixel-based measures of pharyngeal area at maximum constriction, and the ratio of this measure to area at rest, and explored the association between these measures and post-swallow residue using the normalized residue ratio scale (NRRS). Videofluoroscopy data for 5 ml boluses of 22 % (w/v) liquid barium were analyzed from 20 healthy young adults and 40 patients with suspected neurogenic dysphagia. The frames of maximum pharyngeal constriction and post-swallow hyoid rest were extracted. Pixel-based measures of pharyngeal area were made using ImageJ and size-normalized using the squared C2–C4 vertebral distance as a reference scalar. Post-swallow residue and the areas of the vallecular and pyriform sinus spaces were measured on the hyoid rest frame to calculate the NRRSv and NRRSp. The dataset was divided into swallows with residue within or exceeding the upper confidence interval boundary seen in the healthy participants. Mixed model repeated measures ANOVAs were used to compare pharyngeal area (rest, constriction) and the pharyngeal constriction ratio, between individuals with and without residue. Measures of pharyngeal area at maximum constriction were significantly larger (i.e., less constricted, *p* = 0.000) in individuals with post-swallow residue in either the valleculae or the pyriform sinus. These results support the idea that interventions targeted toward improving pharyngeal constriction have the potential to be effective in reducing post-swallow residue.

## Introduction

Pharyngeal constriction has been proposed as a parameter that may distinguish functional from impaired swallows [1–5]. Although pharyngeal manometry is the gold standard with which to measure the strength of the pharyngeal contraction, it is not readily available in many clinical settings. As an alternative to pharyngeal manometry, Leonard and colleagues developed the ‘Pharyngeal Constriction Ratio’ (PCR), a pixel-based measure made on lateral view videofluoroscopy swallow study (VFSS) frames [2]. The PCR is calculated by tracing the unobliterated area of the pharynx (including the bolus) at the point of maximum constriction during the swallow and then dividing that area by a corresponding area measure when the pharynx is at rest. The convention used by Leonard and colleagues is to measure maximum constriction during a 20 cc swallow of liquid barium, and to measure the pharyngeal area at rest with a 1 mL bolus being held in the mouth, prior to swallow initiation [3].

Studies validating the PCR in healthy young and mature adults as well as individuals with dysphagia have shown that ratios larger than 25 % indicate poor pharyngeal constriction; normative values are reported to fall under 14 % across the adult lifespan [3]. The PCR has been validated with manometry and has therefore been suggested as a surrogate method for measuring pharyngeal strength [4]. Additionally, it has been shown that higher PCR measures, indicating poor constriction, are associated with aspiration [5].

There is relatively little known in the dysphagia literature about the mechanisms that lead to the accumulation of residue in the valleculae and pyriform sinuses after a swallow. Hind and colleagues reported that healthy seniors demonstrated less residue when instructed to perform an effortful swallow [6]. Dejaeger and colleagues used both videofluoroscopy and manometry to study residue in 25 healthy older adults and concluded that tongue driving force, pharyngeal shortening, and pharyngeal constriction all play a role in whether or not residue remains after a swallow [1]. Of these mechanisms, tongue driving force was most closely associated with vallecular residue, while pharyngeal shortening and constriction were associated with residue in the pyriform sinuses. However, despite these apparent relationships, treatments targeting improved tongue driving force have, so far, failed to demonstrate convincing improvements on either vallecular or pyriform sinus residues [7, 8]. One reason for the lack of information regarding factors that contribute either to residue accumulation or healthy bolus clearance may be that the field has historically lacked scales with sufficient resolution to distinguish different degrees of residue severity. A recent continuous pixel-based scale of residue measurement, the normalized residue ratio scale (NRRS) [9], offers a new opportunity to study mechanisms involved in pharyngeal residue in greater detail. The NRRS is a continuous scale that is measured objectively and is normalized to the size of the pharynx using the anatomical scalar of the C2–C4 length [9]. It is measured separately for the valleculae (NRRSv) and the pyriform sinus (NRRSp) housings.

In this study, we explored the relationship between pharyngeal constriction and post-swallow residue in the valleculae and pyriform sinuses. Given recent evidence that kinematic measures of pharyngeal swallowing benefit from size-normalization to reduce artifacts attributable to participant height [10], we incorporated anatomical scaling in pixel-based measures of pharyngeal constriction. Our research questions were as follows:

1. What reference values should be used as a basis for determining when measures of pharyngeal constriction are normal or abnormal during a 5 cc thin liquid swallowing task?
2. Do anatomically scaled measures of pharyngeal constriction differ in swallows that display post-swallow residue compared to those with good bolus clearance?
3. Is there a relationship between the degree of pharyngeal constriction seen in a swallow and the severity of post-swallow residue accumulation?

We hypothesized that swallows with a high degree of maximum pharyngeal constriction would show less residue than swallows characterized by poor pharyngeal constriction.

## Methods

### Participants

This study involved retrospective analysis of pharyngeal constriction and residue from videofluoroscopy recordings of thin liquid barium swallows from two previous studies. The first of these studies [11] involved 20 healthy young adults (10 female, 10 male; all under 40 years old), each of whom swallowed three 5 ml boluses of 22 % (w/v) liquid barium. The second [12] was a dataset of 40 patients referred for VFSS due to suspected neurogenic dysphagia (11 female, 29 male; mean age 62, range 18–92). Each patient participant swallowed up to 5 teaspoon-sized boluses of 22 % (w/v) barium. A full description of the methods of both prior studies can be found in [11, 12].

### Data Analysis

**Fig. 1:**
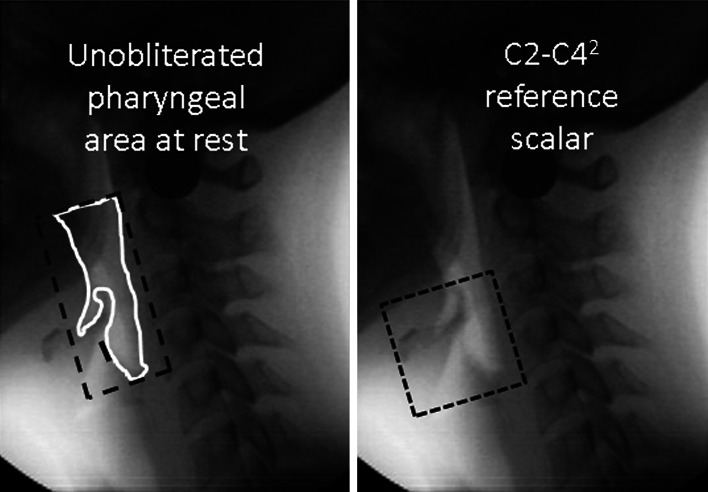
Lateral videofluoroscopic images taken after the hyoid has returned to rest. The *left*-hand image shows a tracing of the unobliterated pharyngeal area in *white*, with the boundaries used for pharyngeal area tracing shown in the *dashed black line*. The *right*-hand image illustrates the anatomical reference scalar used for normalizing measures of pharyngeal area. Normalized area measures can be interpreted as reflecting a percentage of this scalar reference area

VFSS recordings were spliced into individual bolus clips and reviewed frame by frame by two trained research assistants to identify two key frames of interest for the initial swallow of each bolus. The frame of maximum pharyngeal constriction (MPC) was defined as the frame with the smallest amount of unobliterated air space and bolus visible in the pharynx. The frame of post-swallow hyoid rest (HR) was defined as the earliest frame following upper esophageal sphincter closure when the hyoid was observed to have descended and moved posteriorly to reach its original, pre-swallow position. In the event that there were multiple swallows for a bolus, the frame of lowest hyoid position prior to initiation of the first clearing swallow was used as the HR frame. Once these key frames had been indexed, pixel-based measures of pharyngeal area were made using ImageJ software (National Institutes of Health, Bethesda, MD). The boundaries for tracing the unobliterated pharyngeal area are illustrated in Fig. 1 and were defined superiorly as a line perpendicular to the spine connecting the top of the C2 vertebrae to the tongue base; inferiorly as the base of the pyriform sinuses; posteriorly as the pharyngeal wall; and anteriorly as the wall formed by the base of tongue and pharyngeal surface of the epiglottis. In the region of the laryngeal aditus, the anterior margin of the pharynx was traced as a line connecting the base of the epiglottic petiole to the corniculate cartilage at the tip of the arytenoids. (It should be noted that this convention differs slightly from previously described methods of pharyngeal area tracing [2] by excluding the region anterior to the laryngeal aditus.) To correct any differences in pharyngeal size across participants, the pharyngeal area measurements were normalized using an anatomical scalar (the squared length of the C2–C4 vertebral distance). As such, the normalized area measures can be interpreted as a percent of this scalar area, which is shown by the dashed-line square in the right-hand image of Fig. 1. These normalized area parameters will be denoted using a subscript N: normalized maximum pharyngeal constriction area (MPCA_N_) and post-swallow hyoid rest pharyngeal area (HRA_N_). A pharyngeal constriction ratio (PCR_N_) was calculated by expressing the MPCA_N_ as a percentage of the HRA_N_ measure.

In addition to measuring pharyngeal area, residue was measured on the post-swallow hyoid rest frame using the NRRS. Separate measures of residue were taken for the valleculae (NRRSv) and the pyriform sinuses (NRRSp). Inter-rater reliability was tested for each parameter on a random selection of 10 % of the swallows, using two-way mixed intra-class coefficients (ICC) for consistency. Inter-rater agreement was strong, as indicated by intra-class correlations of 0.94 for pharyngeal area measurements on the hyoid rest frame and 0.82 for pharyngeal area measurements on the frame of maximum pharyngeal constriction. Inter-rater agreement for NRRS measures has been reported previously by our group [11].

### Statistical Analysis

Our analysis began by dividing the entire study sample into two residue-severity groups based on the NRRS scores seen on the post-swallow rest frame. First, the means and 95 % confidence intervals for residue were calculated for the healthy controls. NRRSv scores for residue in the valleculae had a mean value of 0.01 and an upper 95 % confidence interval of 0.02 in the healthy control subgroup; this indicates that healthy individuals typically display at most a small coating of residue in the valleculae on 22 % (w/v) thin liquid barium swallows. NRRSp scores for residue in the pyriform sinuses had a mean value and upper 95 % confidence interval of 0.00 in the healthy control subgroup, indicating a complete absence of any pyriform sinus residue. These values were then used to stratify all swallows in the pooled dataset into those displaying “normal” residue (i.e., below the upper 95 % confidence interval boundary for healthy controls), and those with residue exceeding this threshold. Descriptive statistics for pharyngeal area and pharyngeal constriction measures at the swallow level were then explored overall and also by residue-severity grouping. Exploration of the frequency of residue across repeated swallows within an individual led to stratification of the dataset into groupings of participants who displayed residue always, inconsistently, or never (henceforth called “residue pattern”). These stratifications were made separately for vallecular and pyriform sinus residue and used as a between-participants factor for repeated measures mixed model analyses of variance exploring differences in normalized pharyngeal area measurements (HRA_N_, MPCA_N_, PCR_N_) as a function of residue pattern. Effect sizes were calculated using Cohen’s *d*, for which values of 0.2–0.49 are considered small, values of 0.5–0.79 are considered medium and values of 0.8 or higher are considered large [13]. Post hoc analyses of the relationship between MPCA_N_ and NRRS measures were performed using scatter plots, Pearson’s product moment correlations, and linear regression.

## Results

**Fig. 2:**
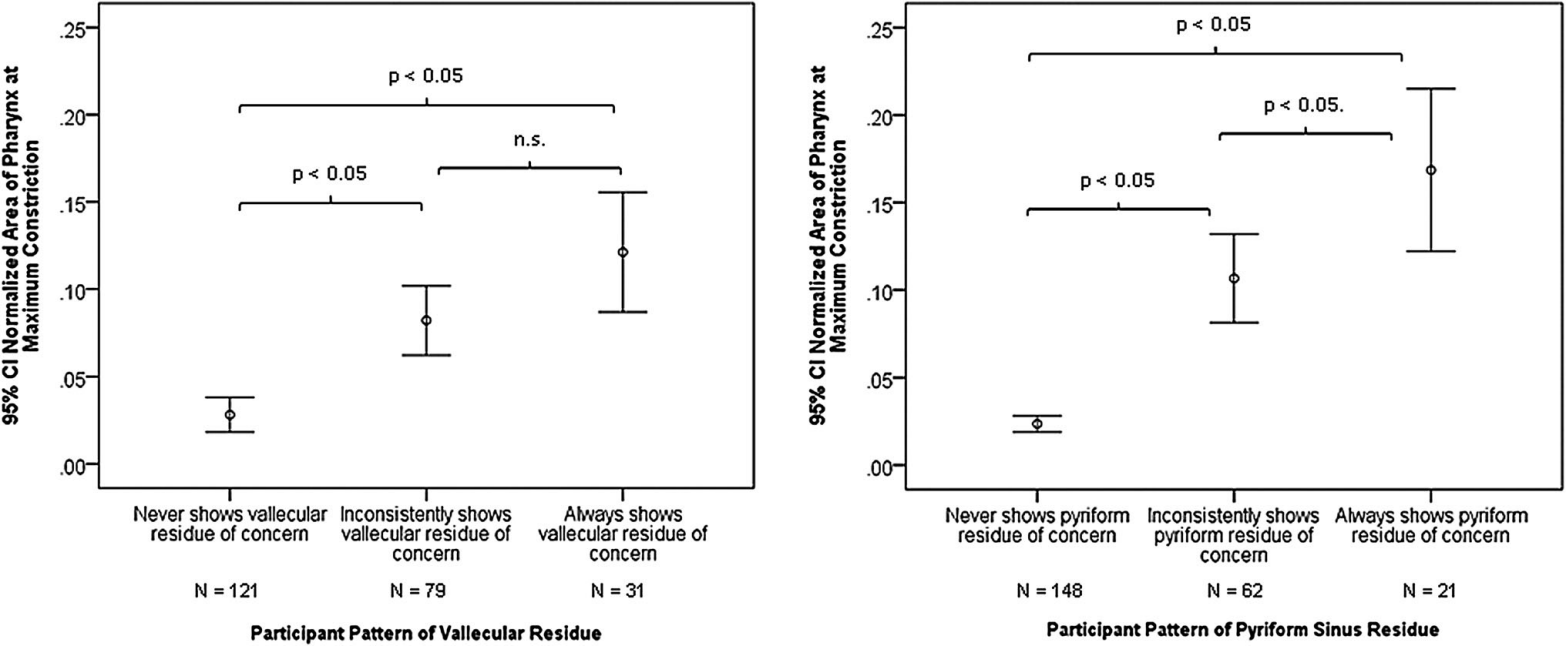
Anatomically normalized measures of maximum pharyngeal constriction area in participants, grouped based on their pattern of displaying post-swallow residue in the valleculae and pyriform sinuses

**Fig. 3:**
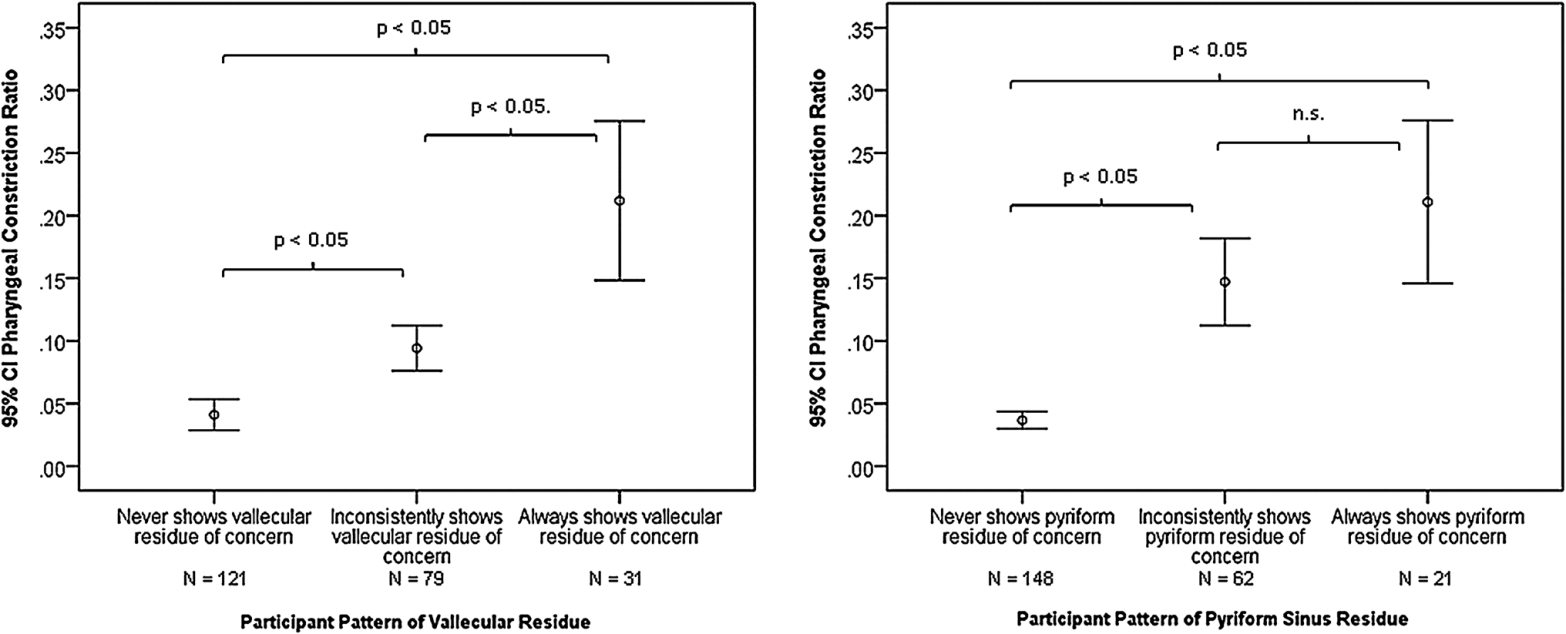
Pharyngeal constriction ratio measures in participants, grouped based on their pattern of displaying post-swallow residue in the valleculae and pyriform sinuses

Descriptive statistics for the pharyngeal area and pharyngeal constriction measures are shown in Table 1. This table lists values for subgroups of swallows in the dataset, depending on whether they displayed residue in the valleculae or pyriform sinuses below or above the 95 % confidence interval boundary seen in the healthy control participants. In total, the dataset contained 231 swallows, of which 65 displayed vallecular residue of concern, and 55 displayed pyriform sinus residue of concern. It can be seen that for the 143 swallows with no residue of concern in either space, the area of the pharynx at maximum pharyngeal constriction was almost completely obliterated, with unobliterated pharyngeal area measures occupying ≤2 % of the scalar reference area. By contrast, swallows resulting in vallecular residue of concern showed less constriction, with 12 % of the scalar reference area remaining unobliterated on average. Swallows resulting in pyriform sinus residue showed an average of 15 % of the scalar reference area unobliterated. Similarly, for swallows without residue, measures of pharyngeal area at post-swallow hyoid rest occupied approximately 2/3 of the anatomical scalar reference area defined by the squared distance of the C2–C4 vertebral length. By contrast, pharyngeal area measures at rest were slightly larger (95 % confidence interval: 70–97 % of the scalar reference area) for swallows displaying residue above the normal limits. When these pharyngeal area measures are entered into the normalized pharyngeal constriction ratio formula, the swallows without residue show almost complete obliteration of the pharynx on maximum constriction, taking up less than 4 % of the corresponding measures at rest. This contrasts with PCR_N_ measures of 14 % or higher in swallows displaying residue of concern in either the valleculae or pyriform sinuses. The mixed model repeated measures ANOVAs confirmed statistically significant differences (*p* < 0.05) in the MPCA_N_ and PCR_N_ measures as a function of residue pattern. There was no significant effect of the repeated swallow factor (*p* = 0.46 for vallecular residue; *p* = 0.11 for pyriform sinus residue). Effect sizes were large, with Cohen’s *d* values of 1.3 and 1.57 for the vallecular and pyriform comparisons of MPAC_N_, respectively, and 1.29 (vallecular) and 1.51 (pyriform) for the comparisons of PCR_N_. These comparisons are illustrated in Figs. 2 and 3. Measures of HRA_N_ between participants with and without vallecular residue approached, but narrowly missed statistical significance (*p* = 0.053) and displayed a small effect size (Cohen’s *d* = 0.29). However, when analyzed with relation to pyriform sinus pattern, a statistically significant main effect and medium effect size was found with smaller HRA_N_ measures in participants who did not display pyriform sinus residue of concern, *F*(2, 66.38) = 3.5, *p* = 0.04; Cohen’s *d* = 0.54.

**Table 1:** Means and 95 % confidence intervals for measures of residue and pharyngeal constriction

	Swallows displaying residue within normal limits (*N* = 143)		Swallows displaying vallecular residue above normal limits (*N* = 65)		Swallows displaying pyriform sinus residue above normal limits (*N* = 55)	
	Mean	95 % confidence interval	Mean	95 % confidence interval	Mean	95 % confidence interval
Vallecular residue (NRRSv)	0.01	0.00–0.02	0.16	0.1–0.23	N/A	N/A
Pyriform sinus residue (NRRSp)	0.00	0.00–0.00	N/A	N/A	0.27	0.13–0.4
Normalized area on frame of maximum pharyngeal constriction (MPCA_N_)	0.02	0.015–0.022	0.12	0.1–0.14	0.15	0.12–0.18
Normalized area on frame of post-swallow hyoid rest (HRA_N_)	0.67	0.63–0.72	0.76	0.7–0.83	0.86	0.74–0.97
Pharyngeal constriction ratio (PCR_N_)	0.03	0.025–0.036	0.17	0.14–0.21	0.19	0.15–0.22

**Fig. 4:**
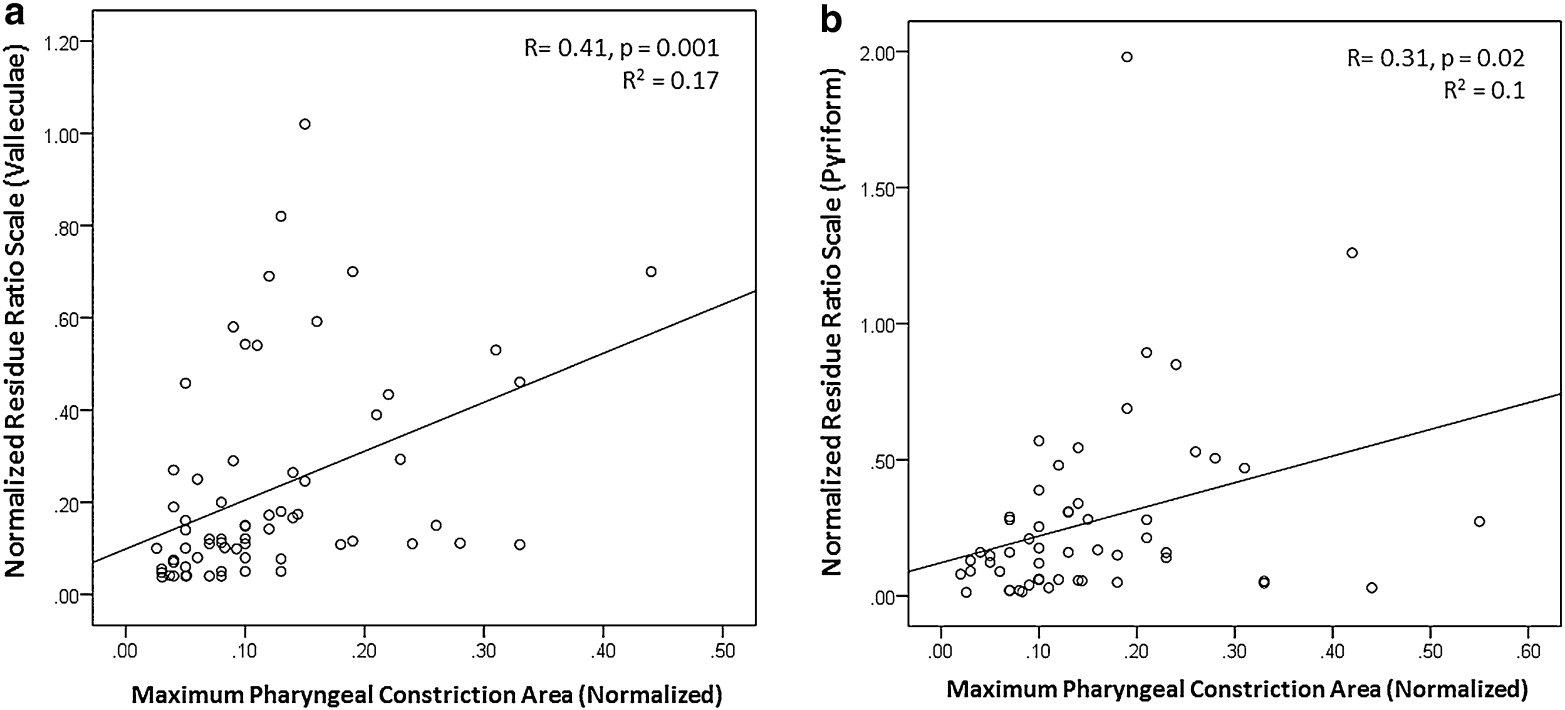
**a** Scatter plot illustrating the severity of post-swallow residue in the valleculae as a function of maximum pharyngeal constriction. **b** Scatter plot illustrating the severity of post-swallow residue in the pyriform sinuses as a function of maximum pharyngeal constriction

Post hoc explorations of the relationship between pharyngeal constriction and residue were performed at the swallow level for all swallows in which residue of concern was seen. Figure 4a, b illustrates this relationship. Although the correlations are weak to modest, the *R* ^2^ values show that the degree of pharyngeal constriction explains 10 % of the variation seen in pyriform sinus residue and 17 % of the variation seen in vallecular residue measures. The overall trend is that larger MPCA_N_ measures (reflecting poorer constriction) are seen for swallows that result in greater accumulation of post-swallow residue.

## Discussion

The purpose of this study was to explore the relationship between pharyngeal constriction during thin liquid swallows and post-swallow residue accumulation in either the valleculae or pyriform sinuses. The results of this analysis are broadly in agreement with those previously reported by Leonard et al. [2], showing that pharyngeal constriction is usually complete during healthy thin liquid swallows. Leonard and colleagues have previously reported PCR as a unitless ratio measure derived from pixel tracings of the pharynx (measured in cm^2^), without normalization to an anatomical scalar. Using these methods on 20 cc thin liquid barium boluses, they found that mean PCR values for healthy individuals fell at or below 0.14. In our study, we took the additional step of calculating the area of maximum pharyngeal constriction in anatomically scaled units, relative to the squared C2–C4 vertebral distance (see Fig. 1). Our results show that for 5cc boluses of thin liquid barium, healthy swallows are associated with almost complete obliteration of the pharyngeal space and with PCR_N_ measures with an upper confidence interval boundary of 0.04. By contrast, swallows that displayed residue in either the valleculae or pyriform sinuses had PCR_N_ values with a lower confidence interval boundary of 0.14, consistent with the numbers reported by Leonard et al. [2].

Our data show that significant differences exist in the amount of visible unobliterated space traceable in the pharynx at maximum constriction between individuals with and without post-swallow residue. Differences in the area of the unobliterated pharynx at rest also approached significance, with larger areas traced in those with pyriform sinus residue, but the effect was not strong. These findings suggest that rather than requiring measurement on two frames and the calculation of a ratio between constricted and unconstructed pharyngeal area, it may be sufficient to determine the adequacy of pharyngeal constriction with a single measurement taken on the frame of maximum constriction. Our data suggest that on thin liquid boluses of 5 cc, this MPCA_N_ measure falls below 3 % of the squared C2–C4 scalar reference area. It should be acknowledged that the normative references in this study are taken from healthy adults under 40 years of age. Leonard and colleagues have previously reported data suggesting that the pharynx may be both longer and larger in seniors [14], perhaps reflecting dilation due to age-related atrophy of the pharyngeal musculature. It would be desirable for similar measures to be established in a control group of healthy seniors to clarify the impact of age versus impairment on pharyngeal constriction.

Notwithstanding the conclusion that ratio measures may not be needed to determine the adequacy of pharyngeal constriction, the finding that participants with residue also trended toward larger anatomically normalized measures of pharyngeal area at rest is notable. The results of the current study suggest that there may be some patients who experience pharyngeal muscle atrophy that leads to dilation of the pharynx and larger pharyngeal area measures at rest. This is consistent with observations previously reported by Leonard and colleagues of larger pharyngeal area at rest in healthy older adults [14]. In our clinical experience, this phenomenon of pharyngeal dilation can occur in patients with chronic dysphagia secondary to brainstem stroke as well as in patients with oculopharyngeal muscular dystrophy. It seems plausible that age-related atrophy or atrophy related to other diagnoses might also lead to this presentation. Thus, two-dimensional measures of the pharyngeal area at rest may have clinical value independent of their contribution to measures of constriction adequacy. The data from this study suggest that individuals with normalized pharyngeal rest area measures >0.72 of the C2–C4 reference scalar area may fall into this hypothetical subgroup of individuals with atrophy-related dilation. The degree to which dilation of the pharynx at rest contributes to the likelihood of residue accumulation independently or in conjunction with measures of pharyngeal constriction deserves further study. Dejaeger et al. [1] identified both pharyngeal constriction and pharyngeal shortening as factors relevant for the accumulation of pyriform sinus residue, and also implicated tongue driving force as a factor contributing to vallecular residue. In this study, we did not measure tongue or pharyngeal pressures, and we did not tease apart the vertical shortening of the pharynx from its circumferential constriction, as viewed on lateral fluoroscopic images. Nevertheless, our findings point to an association between incomplete obliteration of the pharynx and post-swallow residual in both the valleculae and the pyriform sinuses. It is worth noting that this finding was found using thin liquid barium stimuli, and that residue accumulation may be an even greater concern with thicker consistencies [15]. Descriptive statistics for the NRRS in either healthy or disordered swallowing are yet to be reported for thicker consistency stimuli. The current observations lay a foundation for the exploration of pharyngeal constriction measures as an outcome measure in intervention studies employing either compensatory maneuvers such as texture modification or rehabilitative techniques such as the effortful swallow. However, it must also be noted that pharyngeal constriction was found to contribute to only 10–17 % of the variance in post-swallow residue measures, suggesting that other mechanisms are also involved. It would be of great interest to determine whether the relationship between pharyngeal area measures and residue is influenced by bolus consistency. To complete our understanding in this area, investigations of the contribution of bolus volume to pharyngeal constriction area and residue across a range of consistencies would be of value.

Our approach to anatomical normalization of pharyngeal area measures adopted a cervical spine reference scalar. Although this is a convention in the measurement of hyolaryngeal kinematics in swallowing, it must be recognized that changes to the cervical spine related to age, disease, or trauma may render cervical spine scalars invalid as proxies for height and size of the system. Molfenter and Steele have explored the correlations between participant height and 13 anatomical scalars visible in the typical lateral view videofluoroscopy in healthy young adults, and shown that there may be alternatives to cervical spine scalars such as measures of the anterior height of the hyoid lamina or tracheal width [10]. Certainly, determining suitable methods for capturing variations in participant size in populations with alterations to the cervical spine would be desirable.

Finally, it is important to acknowledge the limitation that all measures of residue and pharyngeal area in this study were taken using lateral view images, and are therefore two-dimensional representations of a three-dimensional phenomenon. In particular, the NRRS is unable to capture potential differences between the right and left sides of the pharynx with respect to residue accumulation. Whether the NRRS can be adapted for anterior–posterior views of residue accumulation, employing cervical spine scalars visible in that view, is an interesting question to explore in future research.

## Conclusions

Our results demonstrate a relationship between the degree of pharyngeal constriction and post-swallow pharyngeal residue. Importantly, we were able to clearly demonstrate this relationship using measures of pharyngeal constriction taken from a single frame, which being the frame of maximum pharyngeal constriction. We recommend that this is both conceptually and methodologically simpler than needing to compute a ratio comparing frames of rest and constriction. That said, these results also illustrate that pharyngeal constriction is not the only factor involved in explaining pharyngeal residue. This speaks to the complexity of the physiological presentation of patients with neurogenic dysphagia. It does follow, however, that treatments that improve pharyngeal constriction should result in less residue. These results pave the way for future studies to more clearly explore the benefit of specific dysphagia treatment techniques like effortful swallows and the Mendelsohn maneuver for improving pharyngeal constriction and, in turn, leading to better pharyngeal bolus clearance.
